# Automatic Correction of Real-Word Errors in Spanish Clinical Texts

**DOI:** 10.3390/s21092893

**Published:** 2021-04-21

**Authors:** Daniel Bravo-Candel, Jésica López-Hernández, José Antonio García-Díaz, Fernando Molina-Molina, Francisco García-Sánchez

**Affiliations:** 1 Department of Informatics and Systems, Faculty of Computer Science, Campus de Espinardo, University of Murcia, 30100 Murcia, Spain; daniel.bravo@um.es (D.B.-C.); jesica.lopez@um.es (J.L.-H.); joseantonio.garcia8@um.es (J.A.G.-D.); 2 VÓCALI Sistemas Inteligentes S.L., 30100 Murcia, Spain; fernando.molina@vocali.net

**Keywords:** error correction, real-word error, seq2seq neural machine translation model, clinical texts, word embeddings, natural language processing

## Abstract

Real-word errors are characterized by being actual terms in the dictionary. By providing context, real-word errors are detected. Traditional methods to detect and correct such errors are mostly based on counting the frequency of short word sequences in a corpus. Then, the probability of a word being a real-word error is computed. On the other hand, state-of-the-art approaches make use of deep learning models to learn context by extracting semantic features from text. In this work, a deep learning model were implemented for correcting real-word errors in clinical text. Specifically, a Seq2seq Neural Machine Translation Model mapped erroneous sentences to correct them. For that, different types of error were generated in correct sentences by using rules. Different Seq2seq models were trained and evaluated on two corpora: the Wikicorpus and a collection of three clinical datasets. The medicine corpus was much smaller than the Wikicorpus due to privacy issues when dealing with patient information. Moreover, GloVe and Word2Vec pretrained word embeddings were used to study their performance. Despite the medicine corpus being much smaller than the Wikicorpus, Seq2seq models trained on the medicine corpus performed better than those models trained on the Wikicorpus. Nevertheless, a larger amount of clinical text is required to improve the results.

## 1. Introduction

Clinical notes often contain spelling errors due to time and efficiency pressure. Among abbreviations, punctuation errors and other types of noise, misspellings hinder text processing tasks for knowledge extraction such as term disambiguation and named entities recognition. For that reason, automatic detection and correction of misspellings in medical text are essential to clinical decision support and related endeavors [1,2]. Spelling detection and correction are considered from the perspective of non-real words and real words [3]. The former is concerned about misspellings that result in non-existent words, e.g., ‘graffe’ for ‘giraffe’. These errors are usually detected by looking for them in the dictionary, and corrected by calculating the edit distance from similar words [4]. On the other hand, misspellings that result in actual words are referred to as real-word errors [5,6]. For instance, ‘there’ for ‘three’ results in a real-word error after the transposition of two letters. Similarly, grammatical errors are also considered as real-word errors. In this case, the use of dictionaries is an invalid approach and real words must be analyzed regarding their context.

Several methods have been proposed to correct real-word errors based on the analysis of word context. Traditional approaches may implement rules for error detection based on word similarity [7] and compute n-gram frequencies to determine the correct word [8]. For instance, by splitting texts in short sequences and counting their number of occurrences in a corpus, the probability of a word being a real-word error is obtained. However, short n-grams may convey ambiguous meaning, whereas long n-grams are less frequently found. More recently, deep learning (DL) models have been trained to reflect semantic and syntactic features from words. Recurrent neural networks (RNNs), specifically long short-term memory (LSTM) and gated recurrent unit (GRU) cells, memorize relationships between different time steps in an ordered sequence. Thus, RNNs became the structural units of the encoder-decoder system in seq2seq neural machine translation, which has been proposed for grammatical error correction [9]. By contrast, attention mechanisms have replaced LSTM networks in the transformer architecture providing state-of-the-art results [10,11].

In this work, a DL approach based on a seq2seq neural machine translation model has been implemented to correct real-word errors in medical texts written in Spanish. Large amounts of data are required to train the model. Therefore, synthetic erroneous sentences were generated by applying a set of rules. Each rule generated a different real-word error type. Correct sentences were extracted from the Wikicorpus and a collection of medicine corpora containing clinical notes in Spanish. The use of pre-trained word embeddings is also relevant in order to extract as many context features from words as possible [12]. Therefore, Word2Vec and GloVe embeddings has been also used as the input data to the model. 

The rest of the paper is organized as follows. In Section 2, state-of-the-art approaches for real-word error correction are explored. Then, the approach proposed in this work is described in Section 3, including the corpora used to train the model, pretrained embeddings and the developed model. In Section 4, the experiment conducted to validate the model and its results are discussed. Finally, conclusions and future work are put forward in Section 5.

## 2. Related Works

Eight approaches were studied for the purpose of real-word error automatic correction and other related endeavors. The analyzed approaches were applied to texts in English, Basque, Arabic and Spanish, but related works for Spanish text are not common. The methods encountered have been distinguished between rule-based, n-gram counting, noisy channel, neural embedding and DL methods. Traditional works corresponded to rule-based systems and word or n-gram counting. The two first studied approaches are the result of combining the former for detection and the later for correction. However, error diversity is wide and simple methods may have a limited scope. For that reason, the more complex a model is, the more robust it will be against different types of error. Similar to n-gram language models, word embeddings are used for correcting errors by analyzing their context. On the other hand, DL models learn language representations from previous examples. 

Rule-based approaches are the most straightforward solution. Provided that a condition is met, a predetermined action takes place. However, their implementation is not necessarily easy, since precise knowledge, as well as an efficient, ordered and complete set of rules, are required for testing every possible scenario. 

One example of a rule-based system was developed in [7] for error detection. The following rules were implemented for detecting a set of misspelling corrections: edit distance equal to one, missing white space between two words, edit distance equal to two, words phonetically similar, combination of the first two rules and concatenation of multiple words. These rules were applied sequentially; if a rule matched at least one valid suggestion, the rest were not verified and the procedure was stopped. On the other hand, the authors introduced a frequency-based correction method. Candidates were generated by the detection rules. Suggestions were ranked in two ways: taking into account the context of the misspelled word and calculating single word frequency. In the first case, the Carnegie Mellon University (CMU)-Cambridge Statistical Language Modelling Toolkit was used to compute unigram, bigram and trigram probabilities. In the second case, word occurrences were counted in the training corpus. A knowledge base was built from dictionaries containing standard medical terminology. It was intended to remove as many noisy terms as possible, i.e., abbreviations and variant formats, that could mislead counting. Finally, both context and single frequencies were combined for a more precise correction.

In addition, a set of heuristics was applied, as accuracy of corrections would increase for special cases. For instance, suggestions generated by an edit distance equal to one did not follow the main algorithm and were not ranked by the context-sensitive ranking algorithm. Misspellings of adverbs ending with “-ly” were corrected satisfactorily by picking the first candidate in the ranking ending with “-ly”. The use of heuristics suggests the weakness of rule-based systems due to the variety of error nature. 

On the other hand, n-grams consist of sequences of words. A language model assigns probabilities to sequences of words, i.e., the probability of a sequence to occur in a text or corpus. Common n-grams are made of two and three words (bigrams and trigrams), larger sequences may not be often found in the corpus and would result in poorly accurate models. Interestingly, n-grams take into account the context of the error. 

Pratip and Bidyut [8] developed a system based on the local word bigram and trigram. The probabilities of the n-grams allow the simultaneous detection and correction of real words. To obtain the probabilities, each target word is assigned a confusion set. This set is made of words taken from a dictionary, whose minimum edit distance with respect to the target word is equal to one. The target word is also included. Next, the bigrams and the trigram of all the words in the set are formed and their frequencies are obtained using the Brigham Young University (BYU) corpus n-gram counts. Then, n-gram probabilities are computed. Next, the probability of the left bigram as an example of how to calculate an n-gram probability is defined:

(1) $$P\left(W_{j}^{i}|W^{i-1}\right)=\frac{count\left(W^{i-1}W_{j}^{i}\right)}{\sum_{r=0}^{k_{i}}count\left(W^{i-1}W_{r}^{i}\right)}$$

$W_{j}^{i}$ is the word from the confusion set and $W^{i-1}$ is the word to the left of $W_{j}^{i}$. By counting the occurrences of $W^{i-1}W_{j}^{i}$, $P\left(W_{j}^{i}|W^{i-1}\right)$ is obtained. Finally, the score is computed by a linear combination of left, right bigrams and trigram probabilities. To detect real words, some rules are applied. In general, a real word was detected if the score of the target word was below 0.01. This threshold was the estimated confidence value for a real-word error to occur. Because the score of some correct words could be zero, scoring its stemmed form was also considered. Correction is done by replacing the detected error by the candidate with the highest score. At last, the method was tested on a corrupted set of 25,000 words from the Project Gutenberg corpus (http://www.gutenberg.org/ (accessed on 20 April 2021)). To introduce errors in the test dataset, one from every twenty words was selected to generate a set of strings by substituting, adding or deleting characters. Then, a string was picked at random from those valid words. In this way, 50 real-word errors were introduced.

Alternatively, errors may be thought of as the distorted output of a communication channel: the noisy channel distorts the correct word passing through it. In this way, correction is done by passing every word through a model of the noisy channel and identifying the closest output to the error. Correction is estimated by means of Bayesian inference as follows:

(2) $$c^{\prime }=argmaxP\left(x|w\right)P\left(w\right),$$

P(x|w) is the probability that word w will be distorted (i.e., misspelled) as x, whereas P(w) is the probability of w being generated by the source. Estimation c’ is the correct word predicted by the model. P(w) can be calculated by counting the word frequency in the corpus. P(x|w) can be obtained by computing the inverse of the Damerau–Levenshtein distance between w and x. 

Lai et al. [13] propose the noisy channel for spelling correction in medical texts. First, preprocessing was done so as to minimize errors present in the training corpus and avoid later attempts of correcting proper names, email and website addresses by the spell checker. In addition, commonly misspelled terms such as ‘alot’ were localized and corrected. Stanford Named Entity Recognition was used to avoid misclassification of person names as errors. Second, detection of misspellings was done with the help of dictionaries containing standard medical terminology. If a word was not found in the vocabulary, it was considered as a possible misspelling. However, this approach is not valid for detecting real-word errors. Moreover, a list of suggestions was obtained for each misspelling using Aspell. A ranking system based on Equation (2) was applied. The error was corrected if the first suggestion had a score below a threshold value that varied with the misspelling length. 

Among the solutions that have been described so far, only n-grams take advantage of the error context. Nevertheless, the best current option for that is the use of word embeddings. An embedding is a representation of words in a vector space. A vector represents a word and consists of features describing the context of the word in a corpus. Therefore, two close vectors in the space have similar meaning. 

Fivez et al. [14] took advantage of word embeddings for retrieving contextual information and countering the frequency bias of context-insensitive correction methods based on corpus frequency. A word embedding is a vector representation of a word, where each vector entry contains semantic and syntactic information [3]. Their work is focused on correcting non-word errors that had been already provided, therefore, no detection method is described. On the other hand, the authors recommended this method for correcting real-word errors. Candidates selection was done according to a Damerau–Levenstein distance of 2 and the Double Metaphone from Aspell, an algorithm for determining the similarity of two words by analyzing their phonetics. To create the word embedding, a FastText skip-gram model was trained on a 450-million-word corpus using the default parameters and an embedding dimension of 300. Scoring of correction candidates was done by computing the cosine similarity between the candidate word and the context vector. The context vector was obtained by computing a weighted sum of the word vectors surrounding the error. Finally, the score was divided by the Damerau–Levenshtein distance between the candidate and the error. Should the candidate be not be included in the vocabulary, it would be penalized. The misspelling was replaced by the first candidate in the ranking. 

More recently, DL approaches have been explored. They already implemented solutions for such tasks as speech recognition, language translation and text generation in the Natural Language Processing (NLP) field [3]. Not surprisingly, researchers have been studying their performance on the automatic correction of texts. For instance, the seq2seq neural machine translation [15] is one the most successful methods, although it was initially designed for language translation. It consists of an encoder, which maps a sentence from one language to an intermediate vectorized format, and a decoder, which decodes it into a second language. When applied to automatic correction, the source is the erroneous sentence and the target is the correct sentence. The uselessness of a detection method is noticed, since the whole sentence undergoes translation. Nevertheless, the major drawback of language encoders is the requirement of very large amounts of data for the optimization of the model parameters. As a solution, synthetic data is generated by introducing errors in correct sentences. 

In [11] a seq2seq approach was used for error correction in the Basque language. The studied model was the open-source OpenNMT-py, a Seq2Seq architecture based on a self-attention mechanism [16]. A set of rules generated four types of grammatical error: subject-verb discordance, wrong use of verb tenses, misuse of verbal suffixes and verb paradigm. The rules consisted in replacing specific words. To do so, grammatical information was provided by Eustagger, a morphosyntactic analyzer for the Basque language. For example, a verb in the future tense was replaced by its equivalent in the present tense. By applying these rules in different ways, three datasets were created for training the model. In addition to these, a baseline dataset was compiled, where words were replaced randomly, provided that the bigram (previous word, replacement word) existed in the corpus. The first dataset generated by the rules was obtained by randomly applying just one to each correct sentence. The second dataset contained sentences with one type of error by applying every rule to each correct sentence. At last, the third one contained sentences with several types of error by applying all rules, if possible, to each correct sentence. Moreover, a development dataset was created automatically following the four strategies. An additional manually reviewed dataset was created, since the rules could sometimes generate correct sentences. Finally, unknown words were treated by Byte-Pair Encoding (BPE) tokenization to avoid the open vocabulary issue. 

On the other hand, ELMo (Embeddings from Language Models) [17], BERT (Bidirectional Encoder Representations from Transformers) [18] and RoBERTa (Robustly Optimized BERT pre-training Approach) [19] are considered the state-of-the-art bidirectional pretrained encoders for language modelling. ELMo learns embeddings that vary according to the context. Therefore, a word may have multiple representations, in contrast to those modelled by fastText [20], GloVe [21] and Word2Vec [22]. BERT learns language representations bidirectionally by randomly masking the tokens of the input texts. RoBERTa is a highly pretrained BERT model. All of them can be fine-tuned for different NLP tasks, such as sentiment analysis and next sentence prediction, by adding a few layers and further training the resulting models. 

The ability of these models to detect and locate grammatical errors was studied in [23]. Error examples were taken from the NUCLE (NUS Corpus of Learner English) dataset consisting of pairs of erroneous and correct sentences. A confusion set of words was developed for each error type. The most frequent error types were selected: article, preposition, link words, noun number and verb form replacements, to name a few. The probability of confusing one correct word by another was calculated based on the frequency of that error occurring in NUCLE. To analyze the extent to which the pretrained encoders identify grammatical errors, probabilistic transformation was used. The method consisted in sampling the error examples collected from NUCLE and introducing them in selected positions, which were determined by a parse tree modeled by a syntactic parser. Thus, separate datasets were built for each error type. Each dataset was made of 10,000 sentences of 10 to 60 tokens from the 1B Word Benchmark. One or two errors were introduced to half of the sentences. The datasets were split into training, development and testing sets by a proportion of 80–10–10, respectively. The following pretrained encoders were evaluated: (1) ELMo, pretrained on the bidirectional language modelling task on the 1B Word Benchmark; (2) BERT-base-uncased, pretrained on masked language modelling and next sentence prediction tasks on 16 GB English text; and (3) RoBERTa, pretrained on 160 GB data. An attention-mechanism layer and a binary classifier layer were added on top of these models, which were then trained on the error datasets, while the pretrained layers remained fixed to study their ability to retain information about the error positions. In this manner, the attention layer was intended to evaluate the identification of the error position in the sentence and the binary classifier would determine the correctness of the sentence. As a result, the contextual layers have a stronger ability to detect and locate errors than the input embedding layers. 

In [24] the authors present a system named Sahah for the automatic spelling correction for dyslexic Arabic text. The proposed tool detects misspelled words by searching for each word in a dictionary and combines a statistical approach by using the prediction by partial matching (PPM) compression-based language model and edit operations to generate possible alternatives for each misspelled word. Then, the correct alternative is chosen using the number of bits required to encode the text using a compression algorithm. In their experiments, the authors first evaluated the accuracy of the system using an Arabic corpus containing errors made by dyslexic writers with 28,203 words, namely, the Bangor Dyslexic Arabic Corpus (BDAC), and then compared the results with those obtained using word processing software and an existing text correction tool called Farasa. The BSK corpus, which combines three representative corpora for the Arabic language, was used to train the model.

Finally, a collocation error correction in Spanish learner corpora is described in [25]. Miscollocations are detected using a frequency-based metric. In particular, a corpus consisting of lists of PoS-tagged n-grams in Spanish is used as a reference. If the frequency of a given collocation in the reference n-gram list is bellow an empirically calculated threshold, then such collocation is considered erroneous. Then, in order to correct the miscollocation different strategies are considered. First, synonyms of the terms used in the original collocation are analyzed to check if they form a valid collocation. Otherwise, one out of three possible correction selection metrics are applied to select among correction candidates generated from the original collocation, namely, affinity metric, lexical context metric and context feature metric. The Spanish learner corpus ‘*Corpus Escrito del Español L2′* (CEDEL2) is used in the experiments to test the performance of the collocation recognition approach and each of the miscollocation correction metrics.

Table 1 summarizes the corpora and methods used in each study. Eight different approaches were studied. A rule-based system might be straight-forward for detecting known error types in the text, however, there exist many exceptions which may be unnoticed by the rules. N-grams attempt to model language by grouping together short sequences of words and take into account near context when correcting errors. However, correction is based on counting the frequency of occurrence of n-grams, therefore, very large corpora are required for this solution to be effective. On the other hand, approaches based on neural networks provide more information about context than n-grams, although the issue of the amount of data persists. Finally, most of these methods were applied to English corpora. In this work, neural networks were proposed to automatically correct clinical texts in the Spanish language.

## 3. Seq2Seq Neural Machine Translation-Based Approach to Texts Correction

**Figure 1:**
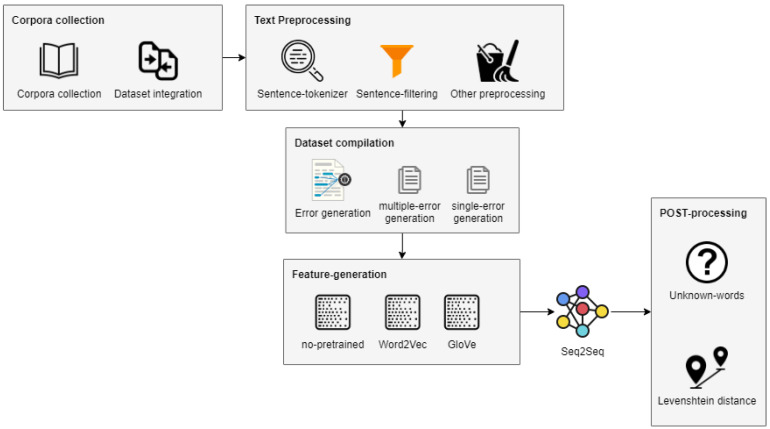
Block diagram of the seq2seq-based approach to clinical texts correction.

In this work we propose the use of seq2seq for Neural Machine Translation. In this way, erroneous texts must be tokenized in sentences, which are the input to the model. The model then maps the erroneous sentences to the correct sentences, provided that it learnt context from previous examples. Additionally, pretrained embeddings containing semantic features have been implemented in the model. The overall process and stages adopted in this research are depicted in Figure 1. In a nutshell, the data analysis pipeline of our proposal is as follows. First, two corpora are collected to train and test the system. For that, different datasets are gathered and integrated. Next, a number of text preprocessing steps are executed in order to remove unwanted characters and symbols. Then, two datasets for each corpus are generated by using two complementary error generation strategies. The implemented seq2seq model is then trained with the generated datasets by considering different settings including the use of GloVe and Word2Vec pretrained embeddings. Finally, a post-processing stage is required to deal with some issues of the seq2seq model when correcting sentences.

In this section, the proposed approach is described in detail. First, some background on seq2seq neural machine translation is provided. Then, the corpora used to train and test the system are pointed out and the pretrained embeddings are outlined. Finally, the steps taken in building the seq2seq model, including text preprocessing, error generation, dataset compilation, model implementation and correction method, are illustrated.

### 3.1. Seq2Seq Neural Machine Translation

Seq2seq [15,26] is based on an encoder–decoder system that translates input sentences into different ones. Difference in length between the input and the output is achieved by an intermediate vector of fixed-size. This characteristic is desirable in language translation, where the word length varies from one language to another. However, the output length may be the same as the input when correcting sentences with errors. In order to extract features from the sequences, the encoder-decoder system consists of two connected RNNs. First, the encoder RNN maps the input sentence into an intermediate vector. Second, the decoder RNN generates the output sentence from the intermediate vector. The conditional probability of the whole output sentence $y_{1},\;\ldots ,\;y_{T^{\prime }}$ provided the input sentence $x_{1},\;\ldots ,\;x_{T}$ is given by the intermediate vector v and the previous output $y_{1},\;\ldots ,\;y_{t-1}$ as shown next:

(3) $$P\left(y_{1},\;\ldots ,\;y_{T^{\prime }}|x_{1},\;\ldots ,\;x_{T}\right)=\overset{T^{\prime }}{\underset{t=1}{\prod }}p(y_{t}|v,\;y_{1},\;\ldots ,\;y_{t-1})$$

For N sentence pair examples, the encoder and the decoder are jointly trained to maximize this probability. 

It is worth noting that a vector of fixed size might not be long enough to store all the features of the input sentence, especially if the input is long. For that reason, the attention mechanism focuses on those parts of the sequences which are the most significant for predicting the target word [27]. An encoder-decoder with attention works similarly, although this time the output sentence is predicted as a function of a context vector along with the previous output, where the context vector $c_{i}$ only contains information about relevant positions in the source:

(4) $$P(y_{i}|y_{1},\;\ldots ,\;y_{i-1},\;x)=f\left(y_{i-1},c_{i}\right)$$

Attention was originally implemented in [27] by using a bidirectional RNN so as to identify information surrounding the input word. It reads the input sequence $x_{1},\;\ldots ,\;x_{T}$ from left to right and vice versa obtaining the forward hidden states $\vec{h_{1}}\;,\;\vec{h_{2}}\;,\;\ldots ,\;\vec{h_{T}}$ and reverse hidden states $\overset{\leftarrow }{h_{1}}\;,\;\overset{\leftarrow }{h_{2}}\;,\;\ldots ,\;\overset{\leftarrow }{h_{T}}$. Then, the hidden states are concatenated to obtain an annotation. Finally, this annotation vector is used by the decoder to compute the context vector.

#### The Transformer Architecture

The transformer is a state-of-the-art encoder-decoder system which performs in the absence of recurrent layers [16]. These are replaced by feed-forward layers and several attention layers. One encoder layer is composed of a multi-head attention layer and feed-forward layer, whereas the decoder layer is composed of two multi-head attention layers and a feed-forward layer. Scale-dot product attention is used: the dot product of a query vector is computed for a number of vectors called keys and a softmax function is applied to obtain the weights on the values. Multi-head attention is the application of several scale-dot product layers in parallel, so that multiple outputs are considered.

### 3.2. Initial Setup

#### 3.2.1. Corpora

Two corpora have been used to train and test the proposed system for real-word error correction: the Wikicorpus and the medicine corpus. The Wikicorpus is the largest one with more than 611 million words extracted from Wikipedia articles in Spanish, whereas the medicine corpus is a collection of approximately 5750 clinical cases and 2 million words derived from three different corpora. The Wikicorpus served as a first test on general language due to the large amount of data required for the correction systems to work. Therefore, once the system performed satisfactorily on this data, it was then tested on the created medical corpus, a much smaller source although indispensable for the purpose of this work.

**Wikicorpus**. The Wikicorpus [28] contains large portions of the Wikipedia in Catalan, Spanish and English. The current version (V.1.0) contains over 750 million words. The texts have been automatically enriched with linguistic information. They have been annotated with lemma and part of speech information using the open source library FreeLing [29]. In addition, word sense disambiguation and lexical similarity features were added using the Word Sense Disambiguation algorithm [30]. 

Wikipedia contains a large amount of freely available text resulting in an attractive resource for NLP applications. However, this fact also requires an effort to cope with text like redirection, disambiguation and poorly edited pages, which are useless. To that end, articles with no category were filtered by the authors. On the other hand, raw text was extracted from the useful articles. Due to processing problems with the text parser, around 80 million words are still missing for the Spanish corpus. The Spanish corpus is the largest one containing over 611 million of words and 455 tokens per article on average. 

The three corpora (Catalan, Spanish, and English) have been made publicly available in two formats. The first one contains raw text from the extracted articles and the second one contains tokenized text with part of speech information. Articles are differentiated from each other by a header and an ending tag. Depending on the user application, further preprocessing might be needed because of the quality of articles that is characteristic of an open resource.

The Spanish corpus in raw format has been used for the purpose of this work, although the annotated text files may be useful for such tasks as preprocessing and grammatical error generation.

**Medicine corpus**. The medicine corpus is a collection of three datasets containing clinical cases in Spanish: CodiEsp, Medical Document Anonymization (MEDDOCAN) and Spanish Clinical Case Corpus (SPACCC). Table 2 shows the size of these datasets.

CodiEsp is a manually coded corpus of clinical case reports written in Spanish that cover diverse medical specialties. The corpus was developed for the Clinical Case Coding in Spanish Shared Task (eHealth CLEF 2020) to promote the development and evaluation of automatic clinical coding systems for medical documents in Spanish. Clinical coding standardizes medical records for health information management systems so as to perform research studies, monitor health trends or facilitate medical billing and reimbursement [31]. The documents are released in plain text format along with separate code files. These last ones consist of manual annotations on the diagnostic and procedure for each clinical case. There are 1000 clinical cases: the training set is made of 500 documents and the development and test sets are made of 250 each. The total number of sentences and tokens are 16,684 and 411,067, respectively. In addition, a background set of 2751 documents was made available for the CodiEsp challenge to encourage participants to scale their coding systems to larger data collections. For the present task, the background set is joined to the training set in order to increase the number of examples.

MEDDOCAN, on the other hand, contains 1000 clinical cases that were selected manually by a practicing physician. These were enriched with protected health information from discharge summaries and medical genetics clinical records. The corpus was created for the MEDOCCAN: Medical Document Anonymization Track, a challenge devoted to the anonymization of medical documents in Spanish. The corpus has around 33,000 total sentences and 33 sentences per clinical case on average. It has 495,000 words in total and an average of 494 words per clinical case. It has been distributed in plain text, where each case is stored in a single file. The corpus has been divided into the training, development and test sets. The training set contains 500 cases, whereas the other two sets contain 250 cases each. In addition, a background set is present to deter manual corrections and to scale systems to larger data collections, however, it is the same set as the one provided by the CodiEsp corpus, so that it has not been included in the final medicine corpus to avoid duplicates.

Finally, the SPACCC corpus is a collection of clinical cases from SciELO (Scientific Electronic Library Online), a source that contains scientific articles from Latin America, South Africa and Spain. The corpus was released for the second Biomedical Abbreviation Recognition and Resolution (BARR2) track, whose aim was to promote the development of systems that were able to identify and define medical abbreviations, a frequent resource amongst healthcare professionals [32]. The corpus contains 1000 articles that were selected by a clinician according to their similarity to real clinical texts in terms of structure and content. The approximate number of sentences and tokens are 20,900 and 436,000, respectively. Figures were removed in order to extract plain text and those articles having multiple cases were divided into separate ones. The downloaded corpus is not classified into training, development and test sets. No annotation is provided either.

#### 3.2.2. Pretrained Embeddings

In this section, pretraining of GloVe and Word2Vec embeddings is described. Word embeddings are vector representation of words in which similar words tend to have similar representation. Word embeddings can be learned from scratch for a specific task, such as sentence classification, or be learned by using a general-purpose task in mind, such as next-word prediction, and transfer this knowledge to a different task resulting in models that generalize better and that they work best on data that they have not seen during training. Our proposal evaluates two different strategies to learn pre-trained word embeddings as well as the word embeddings that are learnt during training for real-word correction. To do this, we set different parameters needed to be specified according to the algorithm used, yet the vocabulary size, word frequency, context window and vector dimension were the same for the two approaches. Moreover, text was previously preprocessed by masking numbers and removing non-alphanumeric characters.

**GloVe**. GloVe embeddings were created using J. Pennington et al. implementation [21]. Training is performed on global word co-occurrences statistics from a corpus. First, the vocabulary was compiled with words occurring with a minimum frequency in the corpus. Second, the algorithm built the co-occurrence matrix, whose entries are the frequency of word pairs. Third, this matrix is provided to learn the embedding. The context window size was set to five and the embedding size was 512. Words occurring 10 or more times were considered. The rest of the parameters were set to their default. However, the script also provided an evaluation step consisting of word similarity queries, i.e., ‘*A is to B what C is to D*’, no improvements were observed by modifying the hyperparameters.

**Word2Vec (W2V)**. Word2Vec was implemented using Python/Gensim [33], a machine learning library for training large-scale semantic NLP models, representing text as vectors and finding semantically related documents. A word embedding was created with the Word2Vec model class. The training corpus was organized in a list of sentences. Words occurring 10 or more times in text were considered. The vector size was set to 512 for training the Seq2seq model. Finally, a context window of five was thought to capture the information necessary for error correction. Since Word2Vec is an unsupervised learning algorithm, there is not an objective evaluation approach.

### 3.3. Seq2Seq Model Building

#### 3.3.1. Text Preprocessing

Python’s Natural Language Toolkit (NLTK, http://www.nltk.org/ (accessed on 20 April 2021)) [34] has been used at the text preprocessing stage. Text was split into sentences by using the *nltk/sent tokenize* method. The method instantiated a tokenizer pretrained on Spanish texts to mark the beginning and the end of sentences. In this way, a list of sentences of variable length were obtained. However, the method failed to process semicolons. These were usually identified as delimiters and were placed at the beginning of the sentences. For that reason, sentences were processed to remove semicolons at the beginning.

Raw text from the Wikicorpus contained invalid lines that should be removed. Such lines were mostly metadata and header and ending strings separating the articles. These lines were identified and filtered. After applying the sentence tokenizer, some sentences contained external links, the title of related articles and short chains with no meaning. These sequences were no longer than 5 tokens and thus were filtered by their length. Additionally, sentences no longer than 40 tokens were selected, since very long sentences could be difficult to learn by the model. 

For the medicine corpus, text from the 3 datasets were joined in a single file. The clinical cases contained mainly free text and no further preprocessing steps were required. By contrast, MEDDOCAN text files were structured in fields with personal data and then a description of patient history was provided at the end. Fields were removed, so that sentences were extracted from the bulk of the text. After tokenizing the text in sentences, these were not filtered by length, since the amount of data was not large enough. 

Finally, numbers were masked by replacing them with a string constant. Then, non-alphanumeric symbols were substituted by a blank space. At last, punctuation signs were separated by a blank space as stated in [35], otherwise the vocabulary would contain compound terms. The generated sentences contained only words and punctuation signs. Moreover, capital letters remained unmodified, since lower-case words at the beginning of a sentence is another type of real-word error.

#### 3.3.2. Error Generation

A sufficient amount of data are needed to train the seq2seq model, however, such amount of text is not available when it comes to the task of real-word error correction. As a solution, errors were introduced in clean text by using rules as it is done in [36]. Errors were generated straightforwardly in the absence of text annotations and syntactic parsers. For instance, some corpora contain part-of-speech information about words that could be useful to generate grammatical errors. On the other hand, syntactic parsers might be used to introduce the errors in the sentences [11]. 

Among the errors generated, six types were distinguished: (1) grammatical genre, (2) number, (3) grammatical genre-number, (4) homophones, (5) Hunspell-generated and (6) subject-verb concordance. The rules were designed to identify specific substrings in the target word and replace them to generate a new word. No syntactic or morphological information was provided to the rules. Therefore, sentence words were iterated by the rules and the first match was used to introduce an error in the sentence. The new words generated by the rules were searched in a dictionary to determine whether they were real words. The dictionary was compiled from the training corpus. All the errors generated differed as much as three edit operations from the correct word, in other words, three or less characters were deleted or added to the word to obtain the error. Table 3 shows an example of an erroneous sentence generated for each type.

1. Grammatical genre errors were generated by identifying the most common morphological suffixes: *-ero*, *-ano*, *-ado*, *-ino*, *-ido*, *-ico*, *-ito*, *-oso*, *-ario*, *-ento*, *-olo*, etc. In Spanish, as opposed to English, nouns, adjectives, or determiners, have lexical gender and vary their inflection in order to share an agreement in the same clause. If the input word ending matched one suffix listed, then the termination was replaced by the opposite, so that the word genre was modified. The rules were applied to masculine and feminine words in both singular and plural forms.
2. In a similar manner, number errors were generated by identifying and modifying the most common word endings in singular, i.e., *-o*, *-e*, *-a*, *-d*, *-n*, *-l*, *-j* and *-r*, or in plural form, i.e., *-os*, *-es*, *-as*. Singular words were replaced by their plural and vice versa, regardless of their genre.
3. Grammatical genre-number rules were combined to generate both kinds of error in the same word. For instance, singular words had their genre modified first and then the plural form was obtained, whereas plural words were changed to singular and then its genre was modified.
4. Writing down a list of homophone words would have been tedious. For that reason, similar rules to those that have been described so far were defined. The most common homophone words contain the letters *b*, *v*, *ll*, y and *h*, since the pairs *b/v* and *ll/y* are equally pronounced in Spanish, whereas the letter *h* is silent. Therefore, the rules determined first whether these characters were present in the target word and then replaced them by the opposite. If a word started with *h* or this letter followed after *n* or *s*, then it was removed. The inverse rules were also considered.
5. In addition to the previous rules, the method *Hunspell/suggest* [37] was used so as to obtain additional real-word error types. The method took an input word and returned a list of terms that were written in a similar way though they differed in meaning. In this way, the first suggestion was picked from the list. The generation of previous errors was avoided by testing the aforementioned rules before picking a word, so that new error types were obtained.
6. The last type of real-word error generated was concerned with subject and verb concordance. Spanish verbs are divided into three conjugation groups: *-ar*, *-er* and *-ir*. For each group, the terminations of the conjugated verbs were listed for past, present and future tenses in both indicative and subjunctive moods. Only regular verbs were considered. Additionally, the conjugation of the verb *haber* was listed, since it is frequently used as the auxiliary verb in perfect tenses. If the input word was a verb, then it was identified as long as its termination appeared in the list and the person was randomly changed by a different one in the same tense. If the termination was not identified, then the whole word was compared to the conjugation list of *haber*. Conjugation groups were identified in the sentence by using the method *Hunspell/stem* [37], which returns the verb in its infinitive form.

There are many grammatical features that were not considered, such as grammatical genre exceptions. Nevertheless, a large enough dataset was obtained by having considered the most common errors. Additionally, word grammatical genre could have been modified by simply identifying the last characters of masculine and feminine words *-a* and *-o*. However, this choice was considered as ambiguous, since many unexpected errors would have been included in the dataset. For instance, the word *casa* is ineligible in this case, because its genre is invariant. The output term *caso* is a real word, but it has a different meaning. By writing down a list of the most common morphological suffixes, there were fewer rule misuse cases and the type of error in the sentence was also known. Finally, the inverse rules were also considered.

#### 3.3.3. Dataset Compilation Strategies

As described in the previous section, rules were created to generate erroneous sentences. Seq2seq input data was organized in one dataset containing the source or erroneous sentences and a second dataset containing the target or correct sentences. Similar to [11], different datasets were compiled according to two strategies: (1) many sentences with different errors were generated from one correct sentence; and (2) only one erroneous sentence was generated from one correct sentence.

1. The first strategy (hereafter, strategy 1) consisted in generating multiple erroneous sentences from one correct sentence. For that, the whole set of rules was selected and applied separately to the correct sentence. In order to balance the number of error types in the dataset, rules were divided according to the error type they generated and, for each group, only one rule was applied at random. Therefore, one sentence or none was obtained for each error type, depending on the rules that were applicable to the correct sentence. Not every attempt succeeded in generating one erroneous sentence. Sentences contained only one error. Additionally, the correct sentence was added to the erroneous dataset [11], as doing so augmented the training data.
2. The second strategy (hereafter, strategy 2) generated only one erroneous sentence from the correct sentence by applying only one rule. Thus, only one error type was generated in the sentence. Again, rules were classified according to the output error type. The groups were applied at random, so as to make sure that the resulting dataset had a balanced number of error types. If the rule generated a real word in the sentence, then the erroneous sentence was added to the dataset and the rest of the rules were not tested. On the other hand, the correct sentence was added to the erroneous dataset only when no rule was able to generate an erroneous sentence.

Rules took single words as input. Therefore, the sentence was split into tokens and iterated by each rule. If a rule matched a token and returned a real word, the matched token was replaced and the resulting sentence was added to the dataset. Errors were likely to be generated at the beginning of the sentence. Moreover, some words occurred quite frequently in the text. In this way, the same error would have been generated often in the error dataset. Such words were listed, identified and ignored before testing the rules to increase error variability. For instance, determinants and prepositions were included in this list. 

These strategies were used to compile four datasets from the medicine and Wikicorpus. The total number of extracted sentences was 103,959 and 3,539,199, respectively. Table 4 and Table 5 show the number of sentences generated for each error type: grammatical genre (E1), number (E2), grammatical genre-number (E3), homophone (E4), Hunspell-generated (E5) and subject-verb concordance (E6). ‘Strategy 1’ and ‘Strategy 2’ stand for the first and second dataset generation strategies described above. Although balance between error types was intended by generating them at random, some of them were obtained more frequently. For instance, it was easier to modify the number of any word than a homophone occurring in the sentence.

#### 3.3.4. Model Implementation

Seq2seq was implemented by using OpenNMT-py, a framework based on Pytorch for implementing seq2seq neural machine translation models [35]. State-of-the-art models can be implemented with OpenNMT-py. LSTM (Long Short-Term Memory), GRU (Gated Recurrent Unit), SRU (Simple Recurrent Unit) hidden units, as well as their bidirectional variants are available for recurrent layers. Additionally, attention mechanisms at decoder level improves context learning on longer sentences. 

Similar to [11], the transformer model was implemented in this work. However, this model differs from [16] in the use of recurrent layers instead of feed-forward layers. Parameter setting was the same, however. For that, see the YAML file in Appendix A. 

OpenNMT-py provides complete documentation and examples of basic scripts for building, training and testing seq2seq models. Parameters for building the vocabulary and training the model were configured in a YAML file. In general, three steps are followed when using OpenNMT-py: vocabulary compilation, training and translation. 

First, vocabularies for both source and target languages were created. For the purpose of error correction one vocabulary was compiled. The vocabulary size may vary between 10 and 50,000 in medium corpora. Large vocabularies complicate learning, whereas small ones may cause translation issues. The number of sample lines from the text is specified to build the vocabulary.

Sentences might be preprocessed before training the model, such as removing words with low frequency and pruning translated sequence length. Similarly, the maximum length of source and target sentences is optionally set. For the error correction task, the input and output length should be the same. Additionally, other sorts of data transformation were available, i.e., data augmentation by random substitution of words, token masking and BPE-dropout [38]. Previous preprocessing was already applied to the Wikicorpus and the medical dataset, so none of these techniques were applied. 

Second, training starts after compiling the vocabulary and preprocessing the data. Both the encoder and the decoder have an embedding layer to learn vector representations of the source and target words. GloVe and Word2Vec pretrained embeddings are also supported. Three seq2seq architectures are available in OpenNMT-py: RNN-based, CNN-based and the transformer. The transformer was proved to learn long-term dependencies between data better than RNN-based models [16]. On the other hand, the CNN-based architecture is not completely implemented. For these reasons, the transformer architecture was chosen along with a recurrent layer as in [11]. The transformer does not support bidirectional recurrent layers, since this feature is only available for the RNN-based architecture. Multiple GPUs might be optionally configured, otherwise training on a single CPU can be very slow. 

Third, translation consisted in predicting correct sentences from erroneous sentences. An option allows the user to replace the unknown words in the predictions with the source token having the highest attention weight. However, such tokens were processed later by comparing them with the source sentence. The minimum and maximum lengths of the prediction can be also optionally set. Finally, ensemble prediction with several models is also possible.

#### 3.3.5. Correction Method

The seq2seq model had some issues when correcting sentences. The development and test sentences were different from the set used to train the seq2seq model. In this way, some words in the test set were not found in the training vocabulary. This issue was treated by writing the constant string <unknown> in the predicted sentence. On the other hand, an unexpected word was sometimes found at the position of a correct word. Furthermore, source and predicted sentences varied in length in some cases. 

Unknown words were treated by replacing them by the source word. Additionally, the Levenshtein distance between the source and the predicted words was measured in an attempt to minimize unexpectedly predicted words. Therefore, the minimum number of edit operations on the original term to obtain the predicted term was calculated. Recall that errors were previously generated by addition and deletion of characters. For that reason, the Levenshtein distance was calculated by taking into account only these edit operations. Next, a threshold distance was set to determine whether to add the source or the predicted token to the correct sentence. If the edit distance was below the threshold, then the prediction was added to the correct sentence. A high threshold increased the possibility of adding a bad prediction to the correct sentence, whereas a low value dismissed the correction of errors with higher distance to the correction. The edit distance to the correct word was 3 for genre, number, genre-number, homophone and Hunspell-generated errors, whereas it was 6 for subject-verb concordance errors.

## 4. Evaluation

The seq2seq model was evaluated with the medicine corpus and the Wikicorpus. The total number of sentences was 103,959 and 3,539,199, respectively. Both sets were also combined in a third set in order to evaluate the model performance in mixed corpora. Recall (R) (Equation (5)), precision (P) (Equation (6)) and $F_{0.5}$ (Equation (7)) were obtained in order to evaluate the seq2seq models. These are the most common measures for the task of real-word error correction [10,11,13].

(5) $$R=\frac{Corrected\;errors}{Total\;errors}$$

(6) $$P=\frac{Corrected\;errors}{Corrected\;errors+Bad\;corrections}$$

(7) $$F_{0.5}=\frac{1.25*P*R}{0.25*P+R}$$

For each set of sentences, errors were generated by following the two aforementioned strategies to compile two training datasets for each corpus as described in Table 4 and Table 5 in Section 3.3.3. The validation dataset used to prevent overfitting contained 5000 sentences. For each corpus, 10,000 sentences were used in total to compile the evaluation dataset, which contained the same number of error types. Seq2seq models were also evaluated according to each error type. The same evaluation dataset was used for both datasets. Moreover, GloVe and Word2Vec pretrained embeddings were used to train different models. The main statistics of the datasets employed in the different evaluation experiments are shown in Table 6.

The transformer was configured in the same way as in [16], since the model was very sensitive to parameter variation. Both the encoder and the decoder contained 6 layers and the number of heads of the multi-attention layer was 8. The dropout ratio was set to 0.1. Moreover, recurrent layers were implemented in the encoder and the decoder. The recurrent layer contained 512 hidden units. The training batch size was 4096 sentences, and the validation batch size was 8. Adam’s optimizer was used. The network weights were initialized to zero and the initial learning rate was set to 2.0 with 8000 warm-up steps. The models were trained until no improvement in the validation set was observed and the model with the highest validation perplexity was chosen. The complete configuration is available in Appendix A.

### 4.1. Experiments Sample Corrections

For illustrating purposes, in Table 7 several correct and incorrect sample corrections are pointed out. In particular, exemplary sentences generated using strategy 2 (i.e., only one error type generated in each sentence) in the medicine corpus are shown (‘source’), along with the original correct sentence (‘target’) and the seq2seq proposed correction. Examples of successful and failed corrections for each error type are put forward.

### 4.2. Performance on All Error Types

Table 8 shows the evaluation of 18 Seq2seq models on all error types described in Section 3.3.2. The threshold Levenshtein distance to correct one word was set to six, since the distance between the error and the correct word could be as much as this value.

For Wikicorpus, the model trained on the training set generated using strategy 1 with no pretrained embedding outperformed the rest ($F_{0.5}=59.77\%$). For this training set, recall and precision were considerably lower when using pretrained embeddings, especially for GloVe with R=12.79% and P=21.71%. However, this was not the case for the models trained on the training set generated using strategy 2 where Word2Vec and GloVe performance improved. Additionally, the results of the model without pretrained embedding considerably decreased on this dataset (R=23.58%, P=46.04% and $F_{0.5}=38.67\%$). In the case of the medicine corpus, the highest performance was given by the models with no pretrained embedding ($F_{0.5}=60.53\%$ and $F_{0.5}=64.98\%$), followed by those using Word2Vec ($F_{0.5}=50.71$ and $F_{0.5}=58.08$. ). On the contrary, using GloVe embeddings provided once more poor results ($F_{0.5}=17.48\%$ and $F_{0.5}=24.01\%$). At last, the results achieved by the models trained on the mixed corpus showed a decrease in performance as compared to the ones obtained on separate corpora. In this case, using GloVe led to the less significant result so far obtained. In general, models trained on the training set generated using strategy 2 performed better than those trained on the training set generated using strategy 1, whereas higher $F_{0.5}$ measures were obtained for the models trained on medicine sentences.

### 4.3. Performance on Each Error Type

The same models were evaluated on each error type: (1) grammatical genre, (2) number, (3) grammatical genre-number, (4) homophones and (5) Hunspell-generated and (6) subject-verb concordance errors.

1. **Evaluation of grammatical genre error correction**. Table 9 shows the performance of the models on grammatical genre error types. In the Wikicorpus, precision of models without pretrained embeddings was high (86.35% and 83.14%) and corrected around 30% of errors. Models using pretrained embeddings provided lower values. The lowest results were obtained for GloVe with R=13.92% and P=30.20%. Nevertheless, a similar performance to the models without pretrained embedding is observed for Word2Vec in the training set generated using strategy 2 with R=30.57% and P=74.60%. In the medicine corpus, the performance of the models improved with respect to the Wikicorpus. The model without pretrained embedding trained on the training set generated using strategy 2 corrected almost 50% of genre errors. However, GloVe performance decreased in the training set generated using strategy 1 ($F_{0.5}=14.22\%$) and in the training set generated using strategy 2 ($F_{0.5}=26.21\%$). Models with pretrained embeddings improved from the training set generated using strategy 1 to the training set generated using strategy 2 for both corpora.

**Table 9:** Recall (R ), precision (P ) and $F_{0.5}$ for the evaluation of genre error correction.

Training Strategy			R	P	$F_{0.5}$
Wikicorpus	Strategy 1	-	30.52	86.35	63.22
		GloVe	13.92	30.20	24.47
		W2V	21.53	65.93	46.68
	Strategy 2	-	32.16	83.14	63.13
		GloVe	25.09	53.56	43.66
		W2V	30.57	74.60	57.92
Medicine corpus	Strategy 1	-	36.71	81.70	65.62
		GloVe	11.51	15.12	14.22
		W2V	30.85	61.19	51.13
	Strategy 2	-	48.10	84.83	73.59
		GloVe	21.48	27.74	26.21
		W2V	45.75	78.99	68.97
2. **Evaluation of number error correction**. Table 10 shows the performance of the models on number error types. In the Wikicorpus, the model without pretrained embedding corrected 43.01% of errors in the training set generated using strategy 1, whereas it corrected 49.58% in the training set generated using strategy 2. Precision is high for both: 87.81% and 84.18%, respectively. GloVe results were much lower in the training set generated using strategy 1 (R=27.12% and P=45.53%) and the training set generated using strategy 2 (R=39.45% and P=62.66%), whereas Word2Vec performed similar to the model without pretrained embedding (R=48.93% and P=81.42%) in the training set generated using strategy 2. In the medicine corpus, models without pretrained embedding and models with Word2Vec improved when trained on medicine sentences. The model without pretrained embedding shows $F_{0.5}=77.22\%$ in the training set generated using strategy 1 and $F_{0.5}=82.82\%$ in the training set generated using strategy 2. This last model corrected more than 70% of sentences. For both corpora, better results were obtained in the training set generated using strategy 2 than in the training set generated using strategy 1.

**Table 10:** Recall (R ), precision (P ) and $F_{0.5}$ for the evaluation of number error correction.

Training Strategy			R	P	$F_{0.5}$
Wikicorpus	Strategy 1	-	43.01	87.81	72.67
		GloVe	27.12	45.53	40.09
		W2V	37.97	77.00	63.87
	Strategy 2	-	49.58	84.18	73.87
		GloVe	39.45	62.66	56.06
		W2V	48.93	81.47	71.91
Medicine corpus	Strategy 1	-	60.32	83.03	77.22
		GloVe	22.35	27.05	25.96
		W2V	55.67	74.54	69.81
	Strategy 2	-	72.11	86.01	82.82
		GloVe	28.24	31.42	30.73
		W2V	66.57	79.15	76.27
3. **Evaluation of grammatical genre-number error correction**. Table 11 shows the performance of the models on grammatical genre-number error types. In the medicine corpus, the model without pretrained embedding and the model with Word2Vec corrected 53.40% and 50.74% of sentences when trained on the training set generated using strategy 1 and a precision of 78.57% and 70.71% was obtained. GloVe performance was again quite below these values, showing $F_{0.5}=18.13\%$. The results improved after training the models on the training set generated using strategy 2. For the model without pretrained embedding, recall was 70.57% and precision 84.90%. The models trained on Wikicorpus sentences performed lower than when trained on medicine sentences. However, this is not true for GloVe in both the training sets generated using strategy 1 and strategy 2, where $F_{0.5}$ was equal to 30.49% and 45.75%, respectively.

**Table 11:** Recall (R ), precision (P ) and $F_{0.5}$ for the evaluation of genre-number error correction.

Training Strategy			R	P	$F_{0.5}$
Wikicorpus	Strategy 1	-	35.07	82.37	64.87
		GloVe	19.45	35.53	30.49
		W2V	30.19	67.61	54.18
	Strategy 2	-	39.61	78.84	65.81
		GloVe	29.97	52.69	45.75
		W2V	38.14	71.60	60.91
Medicine corpus	Strategy 1	-	53.40	78.57	71.80
		GloVe	16.40	18.63	18.13
		W2V	50.74	70.71	65.55
	Strategy 2	-	70.57	84.90	81.59
		GloVe	27.64	30.48	29.87
		W2V	64.94	77.74	74.80
4. **Evaluation of homophone error correction**. A good performance was obtained for the models without pretrained embeddings (see Table 12). In the medicine corpus, recall was equal to 72.24% and precision 88.76% in the training set generated using strategy 1 and 75.68% and 83.30% in the training set generated using strategy 2. In the Wikicorpus, recall was 78.41% and precision was 93.34% when trained on the training set generated using strategy 1, which resulted in $F_{0.5}=89.92\%$. Nevertheless, these values sharply decreased in the training set generated using strategy 2, resulting in $F_{0.5}=42.28\%$. Then, Word2Vec performed well for the medicine corpus in both the training sets generated using strategy 1 and strategy 2 with $F_{0.5}=75.28\%$ and $F_{0.5}=74.34\%$, respectively. Very bad results were obtained for GloVe. For the Wikicorpus, this model corrected less than 9% of sentences in the training set generated using strategy 1 and precision was 19.63%. In line with this, for the medicine corpus this model corrected 26.11% of the sentences and the precision was 26.54%.

**Table 12:** Recall (R ), precision (P ) and $F_{0.5}$ for the evaluation of homophone error correction.

Training Strategy			R	P	$F_{0.5}$
Wikicorpus	Strategy 1	-	78.41	93.34	89.92
		GloVe	8.77	19.63	15.73
		W2V	13.20	51.16	32.48
	Strategy 2	-	17.20	66.52	42.28
		GloVe	12.98	33.62	25.51
		W2V	30.57	74.59	57.92
Medicine corpus	Strategy 1	-	72.24	88.76	84.88
		GloVe	26.11	26.65	26.54
		W2V	70.75	76.50	75.28
	Strategy 2	-	75.68	83.30	81.66
		GloVe	28.85	29.68	29.51
		W2V	69.66	75.63	74.34
5. **Evaluation of Hunspell-generated error correction**. In Table 13, for the medicine corpus the model without pretrained embedding corrected only 36.10% of sentences in the training set generated using strategy 1, whereas the model with Word2Vec corrected 30.08%. However, the precision of the former is 75.40% compared to 56.13% of the latter. In the Wikicorpus, the number of corrected sentences decreased to 17.42% for the model without pretrained embedding in the training set generated using strategy 1. The three models revealed a very bad performance in the training set generated using strategy 2. Actually, for this error type, results decreased in all models that were trained on the training set generated using strategy 2 in both corpora.

**Table 13:** Recall (R ), precision (P ) and $F_{0.5}$ for the evaluation of Hunspell-generated error correction.

Training Strategy			R	P	$F_{0.5}$
Wikicorpus	Strategy 1	-	17.42	78.06	46.03
		GloVe	15.49	40.96	30.82
		W2V	20.27	58.74	42.58
	Strategy 2	-	10.25	56.60	29.72
		GloVe	7.17	28.00	17.71
		W2V	7.52	31.88	19.34
Medicine corpus	Strategy 1	-	36.10	75.40	61.92
		GloVe	13.31	16.36	15.64
		W2V	30.08	56.13	47.85
	Strategy 2	-	20.60	54.57	41.04
		GloVe	9.69	12.95	12.13
		W2V	20.65	44.46	36.13
6. **Evaluation of subject-verb concordance error correction**. Models show better performance when trained on the medicine corpus than on the Wikicorpus (see Table 14). In the medicine corpus, the model without pretrained embedding corrected 44.55% of sentences in the training set generated using strategy 1 and 47.39% in the training set generated using strategy 2. Precision was low for both datasets (59.30% and 62.18%). The model with Word2Vec corrected 41.37% of sentences in the training set generated using strategy 1 and 43.01% in the training set generated using strategy 2, whereas the model with GloVe corrected 16.65% of sentences in the training set generated using strategy 1 and 19.72% in the training set generated using strategy 2. The results of the models in the Wikicorpus are poorer than in the medicine corpus. The model without pretrained embedding corrected 24.55% of sentences in the training set generated using strategy 1, whereas the models using GloVe and Word2Vec corrected 7.89% and 10.96%, respectively.

**Table 14:** Recall (R ), precision (P ) and $F_{0.5}$ for the evaluation of subject-verb concordance error correction.

Training Strategy			R	P	$F_{0.5}$
Wikicorpus	Strategy 1	-	24.55	50.00	41.41
		GloVe	7.89	16.55	13.57
		W2V	10.96	33.95	23.92
	Strategy 2	-	15.23	44.48	32.14
		GloVe	11.89	32.58	24.17
		W2V	14.24	38.46	28.70
Medicine corpus	Strategy 1	-	44.55	59.30	55.61
		GloVe	16.65	18.69	18.25
		W2V	41.37	52.07	49.51
	Strategy 2	-	47.39	62.18	58.53
		GloVe	19.72	22.74	22.06
		W2V	43.01	55.59	52.52

The results obtained from the evaluation of subject-verb concordance error correction were very poor. The reason for that could have been the large Levenshtein distance between the generated error and the correct word, which in some cases was as much as six. Similarly, some Hunspell-generated errors had distances larger than three. Moreover, mistakes usually occur by editing one or two characters. Errors with a higher number of edit operations are unlikely. Therefore, new models were trained on sentences without subject-verb concordance error, whereas Hunspell-generated errors of distance equal to two were selected. The goal was to find out whether correction results improved.

Table 15 shows $F_{0.5}$ for (E1) grammatical genre, (E2) number, (E3) grammatical genre-number, (E4) homophones and (E5) Hunspell-generated errors. These values were compared before and after removing subject-verb concordance error types. Results were obtained for both Wiki and medicine corpora.

### 4.4. Discussion

Compared to [11], in which the authors use seq2seq for grammatical error correction for the Basque language, the performance of seq2seq for the corpus used in this research was low. Taking into account all error types, the best model trained on the medicine corpus only corrected 50% of the total number of sentences, whereas precision was near 70%. On the other hand, a few models revealed good results for homophone, number and grammatical genre-number, where the number of corrected sentences increased to 70%. Correction of errors generated with Hunspell and subject-verb concordance errors showed the lowest rate. 

Models trained on the Wikicorpus were expected initially to perform better than those trained on medicine sentences, due to the larger amount of data. Wikicorpus sentences varied in length from 5 to 60 tokens. Moreover, article topics were diverse, which resulted in a very large vocabulary and unknown words in the evaluation set, whereas known words were used in different contexts. At last, Wikipedia is open source and widely contributed, so that they may contain syntactic and grammatical errors. On the contrary, clinical text vocabulary and context was reduced to one single domain and sentences were written in a simple style. This could answer why results improved for models trained on the medicine corpus even if the dataset was smaller. 

After comparison of the models trained on the training set generated using strategy 1 to those trained on the training set generated using strategy 2, different results were obtained according to the type of error. In general, models performed better on the training set generated using strategy 2 for genre, number and grammatical genre-number errors, whereas better results were obtained on the training set generated using strategy 1 for Hunspell-generated errors. Repeating the same sentence with a different error each time might be adequate depending on the error type. Grammatical genre and number variants of a same word convey the same meaning in a sentence, although it is not grammatically correct. In fact, these categories would be produced in the training set generated using strategy 1 by modifying the grammatical genre and number of the same word to form different sentences. This would lead to repeating data and, thus, overfitting. However, Hunspell-generated errors completely differed in meaning. In this way, the modified sentence was different with respect to the others, providing the model with a new example. Nevertheless, different models should be trained on Hunspell-generated errors only and then grammatical genre and number errors to clarify the statement of data redundancy. 

The use of GloVe and Word2Vec pretrained embeddings had different impacts on the results. These approaches extract features by means of different techniques. Nevertheless, the fact that the lowest results were obtained by GloVe does not imply that this approach is less suitable for learning embeddings. On the other hand, success is believed to rely on the adjustment of parameters. The model is not sensitive to the variation of every parameter, therefore, only some of them may affect the results. The embedding size used in the experiments was 512, however, a common value is 300. The transformer required this parameter to be equal to the number of units in the recurrent layers of the encoder and the decoder. By decreasing the size of the recurrent layers, the performance did as well, due to the sensibility of the transformer architecture. Therefore, whether the embedding size affected the final result, it should not be modified. On the contrary, Word2Vec performed much better with the same embedding size. For future work, the impact on performance of other related feature extraction techniques such as doc2vec, TF-IDF and bag-of-words (BoW) will be explored.

By combining several corpora, larger datasets could be obtained to train the Seq2seq model. The same number of sentences from the Wikicorpus were added to the medicine sentences so as to determine whether results improved. On the contrary, this solution did not work as performance decreased for all the models. The medicine corpus itself is a collection of three different sources, however, they all belong to the same domain and sentences are written in a similar style. Relating two completely different corpora might have been the reason for a decrease in the results. 

By removing the subject-verb concordance errors (Table 15), F0.5 decreased for each error type except for Hunspell-generated errors (E5) and many were removed from the training set generated using strategy 1, decreasing the training dataset size. On the contrary, more errors of the other categories were generated without repetition of sentences and the same dataset size was obtained in the training set generated using strategy 2, yet the results were lower than before. This suggests that error variability in the training dataset improves the performance of seq2seq correction. On the other hand, correction of Hunspell-generated errors improved by selecting those of Levenshtein distance shorter than two.

## 5. Conclusions and Future Work

Real-word errors can affect the performance of automatic text processing tools, including decision support systems and recommender systems. In the medical domain, these errors can even lead to serious deficiencies in patient care services [13]. In order to tackle this problem, in this work a DL approach was evaluated on the task of real-word error correction in Spanish clinical texts. In particular, we proposed the use of a Seq2seq model for neural machine translation, which translates erroneous sentences to correct ones. Moreover, in the absence of sufficiently large amounts of text, the implementation of pretrained embeddings and model performance on mixed corpora were evaluated in this work. 

The performance results obtained were poorer than expected at the beginning, especially considering those published in [11]. It is worth noting that Basque (the language on which [11] is focused) and Spanish are completely different, so the comparison of the results must be taken with caution. First, Basque is an agglutinative language whereas Spanish is fusional, so the way in which words are composed is different and so is the probability of generating real-word errors. Moreover, although Spanish and Basque share the alphabet to a great extent, the conjugations and declinations are very different. Second, Basque is the only surviving Pre-Indo-European language in Western Europe. Spanish, in contrast, is derived from Latin and has a very wide lexical richness with words derived from Italian, French, English, Romanian and Latin America. Third, Basque has only rarely accented markers, which can reduce the possibility of producing real-word errors as with the diacritical mark in Spanish. Forth, Spanish has many homonymy words since the ‘h’ is silent in Spanish and several letters in Spanish are pronounced the same (e.g., ‘b’-‘v’, ‘g’-‘j’, ‘ll’-‘y’).

The medicine corpus was much smaller in size than the Wikicorpus, but context was restricted to a unique domain and the sentences extracted were uniform, which led to better results. By contrast, not enough samples were obtained and clinical text scarcity continues to be an issue. Moreover, repeating several times a sentence by modifying word genre and number may produce model overfitting during training, since genre and number do not change the meaning of the correct word, whereas Hunspell-generated errors completely differ in meaning, which may provide new samples.

Performance clearly decreased in the experiments when using pretrained embeddings. The poorest results were obtained with GloVe, although those obtained with Word2Vec were not far from the best models. A possible explanation for this is the difficulty of training word vectors with a large embedding size. For future work, a new GloVe embedding would be trained on a larger corpus such as the Spanish Billion Word Corpus (SPWC) [39]. In addition, we will check the reliability of contextual word embeddings based on BETO (https://github.com/dccuchile/beto (accessed on 20 April 2021)) [40] and then fine-tune them to the medical domain. We will also evaluate the reliability of pretrained word embeddings like FastText, which, contrary to Word2Vec, also handles out-of-vocabulary words because word embeddings are learned from character n-grams instead of words. These Spanish word embeddings have been evaluated in other NLP tasks with promising results [41,42,43]. Moreover, the possibility to adapt non-Spanish pre-trained embeddings specifically built for domains close to the medical one such as BioBERT (‘Bidirectional Encoder Representations from Transformers for Biomedical Text Mining’, https://github.com/naver/biobert-pretrained (accessed on 20 April 2021)) [44] will be analyzed.

Joining both Wiki and medicine corpora decreased the performance of the models. This solution might be adopted as long as the data sources are similar. Therefore, a model for correcting medical texts should be trained exclusively on data from that domain. In line with this, new training datasets could be compiled in future work, so that they only contain one error type to study the variation of model performance. For instance, different models could be trained on Hunspell-generated errors only and on genre and number errors to study data redundancy.

A natural next step, which we leave for future work, is to assess whether clinical decision support systems improve when dealing with the corrected sentences or not. Similarly, the impact of real-word errors correction in semantic-based recommendation engines such as that described in [45] is to be measured. 

## Figures and Tables

**Table 1:** Corpora and methods used in each study.

Study	Corpora	Methods	Language
[7]	Corpus of clinical records of the Emergency Department at the Concord Hospital, Sidney.	Rule-based system and frequency-based method for detection and correction, respectively.	English
[8]	Brigham Young University bigram and trigram corpus.	Correction frequency-based method: bigram and trigram counting.	English
[13]	Free-text allergy entries from Allergy Repository (PEAR).	Dictionary look-up detection method and noisy channel correction method.	English
[14]	Clinical free-text from the Medical Information Mart for Intensive Care III (MIMIC-III) database.	No detection method. Word embedding cosine similarity for correction.	English
[11]	Sentences extracted from Basque news websites.	Correction with seq-2-seq Neural Machine Translation model.	Basque
[23]	Sentence pairs from 1B Word Benchmark.	BERT, RoBERTa and ELMo encoders and feature/fine-tuning based training for error location.	English
[24]	Bangor Dyslexic Arabic Corpus (BDAC).	Dictionary look-up detection method. PPM language model, edit operations and compression codelength for correction.	Arabic
[25]	*Corpus Escrito del Español L2* (CEDEL2).	Error detection based on the frequency of n-grams. Affinity, lexical context and context feature metrics to select candidate correction.	Spanish

**Table 2:** Size of the Spanish medicine datasets.

Dataset	Cases	Sentences	Tokens
CodiEsp	3751	50,052	1,233,201
MEDDOCAN	1000	33,000	495,000
SPACCC	20,900	20,900	436,000

**Table 3:** Different error types used. Errors were generated by replacing word substrings (in Spanish).

Error Type	Substring Replacement	Erroneous Sentence
Grammatical genre	*-ido* → *-ida*	Dicho traspaso se realiza **unida** al de su compañero.
	*-eras* → *-eros*	Las aguas del oeste son **traicioneros**.
Number	*-e* → *-es*	La **aeronaves** terminó contra un muro de contención.
	*-des* → *-d*	Consciente de sus numerosas **virtud**.
Grammatical genre-number	*-osos* → *-osa*	Se caracterizan por tener diseños agresivos y **sinuosa**.
	*-ado* → *-adas*	El proyecto fue **abandonadas** hace años.
Homophones	*a-* → *ha-*	Es una de las compañías más importantes **ha** nivel mundial.
	*-n-* → *-nh-*	Receta de pollo con lima y **enhebro**.
Hunspell	*calle* → *callé*	Más de la mitad de la gente vive ahora en la **callé**.
	*agravio* → *agrado*	El **agrado** costó el reemplazo de los muebles.
Subject-verb concordance	*-an* → *-as*	Tanto las mujeres como los hombres las **llevas**.
	*-aba* → *-ábamos*	**Peleábamos** con los invitados para divertirse.

**Table 4:** Number of sentences for each error type for the Wikicorpus datasets. Error types: genre (E1), number (E2), genre-number (E3), homophone (E4), Hunspell-generated (E5) and subject-verb concordance (E6).

Wikicorpus	E1	E2	E3	E4	E5	E6
Strategy 1	1,952,506	3,196,193	2,027,014	1,712,850	2,753,397	1,786,375
Strategy 2	472,376	788,092	492,374	415,709	671,898	430,993

**Table 5:** Number of sentences for each error type for the medicine datasets. Error types: genre (E1), number (E2), genre-number (E3), homophone (E4), Hunspell-generated (E5) and subject-verb concordance (E6).

Medicine	E1	E2	E3	E4	E5	E6
Strategy 1	64,439	100,871	71,027	27,609	90,917	67,849
Strategy 2	18,537	24,261	19,704	6000	20,004	18,852

**Table 6:** Evaluation datasets statistics.

	Training	Validation *	Test	Total
**Wikicorpus**				
Strategy 1	14,181,497	5000	10,000	14,196,497
Strategy 2	3,512,147	5000	10,000	3,527,147
**Medicine corpus**				
Strategy 1	276,835	5000	9533	291,368
Strategy 2	75,062	5000	9533	89,595
**Medicine-Wikicorpus**				
Strategy 1	553,670	5000	10,000	568,670
Strategy 2	150,124	5000	10,000	165,124

* The validation dataset is used during training to avoid the model overfitting.

**Table 7:** Sample successful and failed corrections (in Spanish).

Error Type	Result	Sentence (Source/Target/seq2seq Correction)
Grammatical genre	Success	Source: “Se procede a la electrocoagulación de varias áreas con sangrado **activa**” Target: “Se procede a la electrocoagulación de varias áreas con sangrado **activo** “ Seq2seq correction: “Se procede a la electrocoagulación de varias áreas con sangrado **activo**”
	Failure	Source: “Estabilización y **traslada** a planta de medicina interna” Target: “Estabilización y **traslado** a planta de medicina interna” Seq2seq correction: “Estabilización y **traslada** a planta de medicina interna”
Number	Success	Source: “No obstante el **pacientes** se encuentra tranquilo en actitud plácida” Target: “ No obstante el **paciente** se encuentra tranquilo en actitud plácida” Seq2seq correction: “No obstante el **paciente** se encuentra tranquilo en actitud plácida”
	Failure	Source: “Sin **antecedente** de enfermedades cronicodegenerativas” Target: “Sin **antecedentes** de enfermedades cronicodegenerativas” Seq2seq correction: “Sin **antecedente** de enfermedades cronicodegenerativas”
Grammatical genre-number	Success	Source: “Los **cultiva** del LCR fueron negativos” Target: “Los **cultivos** del LCR fueron negativos” Seq2seq correction: “Los **cultivos** del LCR fueron negativos”
	Failure	Source: “La movilidad facial estaba **conservados**” Target: “La movilidad facial estaba **conservada**” Seq2seq correction: “La movilidad facial estaba **conservados**”
Homophones	Success	Source: “Durante un año de seguimiento **a** presentado esputos hemoptoicos ocasionales” Target: “Durante un año de seguimiento **ha** presentado esputos hemoptoicos ocasionales” Seq2seq correction: “Durante un año de seguimiento **ha** presentado esputos hemoptoicos ocasionales”
	Failure	Source: “Tampoco refiere el **huso** de algún fármaco de forma usual ni alergias” Target: “Tampoco refiere el **uso** de algún fármaco de forma usual ni alergias” Seq2seq correction: “Tampoco refiere el **uso** de algún fármaco **en** forma usual ni alergias”
Hunspell	Success	Source: “En la observación **genera**, el abdomen se presentaba blando e indoloro” Target: “En la observación **general**, el abdomen se presentaba blando e indoloro” Seq2seq correction: “En la observación **general**, el abdomen se presentaba blando e indoloro”
	Failure	Source: “Tras la administración de contraste la **amas** realzaba su densidad” Target: “Tras la administración de contraste la **masa** realzaba su densidad” Seq2seq correction: “Tras la administración de contraste la **amas** realzaba su densidad”
Subject-verb concordance	Success	Source: “Se **realizo** ECO doppler y angioma TAC” Target: “Se **realiza** ECO doppler y angioma TAC” Seq2seq correction: “Se **realiza** ECO doppler y angioma TAC”
	Failure	Source: “Se **realizaron** una artrocentesis bajo control de radioscopia con anestesia general” Target: “Se **realizó** una artrocentesis bajo control de radioscopia con anestesia general” Seq2seq correction: “Se **realizaron** una artrocentesis bajo control de radioscopia con anestesia general”

**Table 8:** Recall (R ), precision (P ) and $F_{0.5}$ measure for the datasets generated according to the strategies listed in Section 3.3.3 (‘Strategy 1’ and ‘Strategy 2’). The results are shown for the Seq2seq models with GloVe and Word2Vec pretrained embeddings and with no pretrained embedding at all (-). All error types were considered.

Training Strategy			R	P	$F_{0.5}$
Wikicorpus	Strategy 1	-	37.61	70.19	59.77
		GloVe	12.79	21.71	19.05
		W2V	18.88	39.72	32.54
	Strategy 2	-	23.58	46.04	38.67
		GloVe	21.16	41.65	34.89
		W2V	23.84	48.42	40.15
Medicine corpus	Strategy 1	-	46.06	65.68	60.53
		GloVe	15.79	17.95	17.48
		W2V	40.07	54.31	50.71
	Strategy 2	-	50.92	69.79	64.98
		GloVe	21.58	24.71	24.01
		W2V	46.69	61.82	58.08
Medicine-wikicorpus	Strategy 1	-	20.61	45.83	36.82
		GloVe	3.76	4.61	4.41
		W2V	14.88	24.79	21.88
	Strategy 2	-	27.92	56.33	46.81
		GloVe	10.63	13.36	12.70
		W2V	23.36	40.92	35.57

**Table 15:** Comparison of $F_{0.5}$ measures before and after removing the subject-verb concordance error type and selecting Hunspell-generated errors of Levenshtein distance equal to two. No pretrained embedding was used. (E1) genre, (E2) number, (E3) genre-number, (E4) homophones and (E5) Hunspell-generated errors.

Training Strategy			E1	E2	E3	E4	E5
Wikicorpus	Strategy 1	Before	63.22	72.67	64.87	89.92	46.03
		After	54.41	65.30	50.81	86.91	78.92
	Strategy 2	Before	63.13	73.83	65.81	42.28	29.72
		After	51.29	67.36	49.68	73.74	70.99
Medicine corpus	Strategy 1	Before	65.62	77.22	71.80	84.88	61.92
		After	50.02	71.96	62.14	77.09	77.53
	Strategy 2	Before	73.59	82.82	81.59	81.66	41.04
		After	56.52	74.78	67.16	72.44	74.42

## Data Availability

Publicly available datasets were analyzed in this study. This data can be found here: https://pln.inf.um.es/corpora/realworderrors/datasets.rar (accessed on 20 April 2021). In addition, source code of the experiments can be found here: https://github.com/fgs22002/real-word-errors (accessed on 20 April 2021).

## Appendix A

Seq2seq YAML configuration file:

# Where the samples will be written

save_data: wiki_spanish/run/example

# Where the vocab(s) will be written

src_vocab: wiki_spanish/run/example.vocab.src

tgt_vocab: wiki_spanish/run/example.vocab.tgt

# Prevent overwriting existing files in the folder

overwrite: True

# Corpus opts:

data:

corpus_1:

path_src:/directory/of/the/source/training/set/

path_tgt:/directory/of/the/target/training/set/

valid:

path_src:/directory/of/the/source/development/set/

path_tgt:/directory/of/the/target/development/set/

# Vocabulary files that were just created

src_vocab: directory/of/the/source/vocabulary/

tgt_vocab: directory/of/the/target/vocabulary/

# General opts

save_model:/directory/of/the/saved/model/

save_checkpoint_steps: 500

valid_steps: 500

train_steps: 2000

# Batching

queue_size: 10000

bucket_size: 32768

batch_type: “tokens”

batch_size: 4096

valid_batch_size: 8

max_generator_batches: 2

accum_count: [4]

accum_steps: [0]

# Optimization

model_dtype: “fp32”

optim: “adam”

learning_rate: 2

warmup_steps: 8000

decay_method: “noam”

adam_beta2: 0.998

max_grad_norm: 0

label_smoothing: 0.1

param_init: 0

param_init_glorot: true

normalization: “tokens”

# Model

encoder_type: transformer

decoder_type: transformer

position_encoding: true

enc_layers: 6

dec_layers: 6

heads: 8

rnn_size: 512

word_vec_size: 512

transformer_ff: 2048

dropout_steps: [0]

dropout: [0.1]

attention_dropout: [0.1]

## References

[1] Hussain F., Qamar U., Hammoudi S., Maciaszek L.A., Missikoff M., Camp O., Cordeiro J. (2016). Identification and Correction of Misspelled Drugs’ Names in Electronic Medical Records (EMR). Proceedings of the 18th International Conference on Enterprise Information Systems (ICEIS 2016).

[2] Workman T.E., Shao Y., Divita G., Zeng-Treitler Q. (2019). An efficient prototype method to identify and correct misspellings in clinical text. BMC Res. Notes.

[3] Jurafsky D., Martin J.H. (2020). Speech and Language Processing (Draft).

[4] López-Hernández J., Almela Á., Valencia-García R., Valencia-Garcıa R., Alcaraz-Mármol G., del Cioppo-Morstadt J., Bucaram-Leverone M. (2019). Automatic Spelling Detection and Correction in the Medical Domain: A Systematic Literature Review. Technologies and Innovation, Proceedings of the 5th International Conference, CITI 2019, Guayaquil, Ecuador, 2–5 December 2019.

[5] Ramshaw L.A. (1994). Correcting real-word spelling errors using a model of the problem-solving context. Comput. Intell..

[6] Sharma S., Gupta S. (2015). A Correction Model for Real-word Errors. Procedia Comput. Sci..

[7] Patrick J., Sabbagh M., Jain S., Zheng H. Spelling correction in clinical notes with emphasis on first suggestion accuracy. Proceedings of the 2nd Workshop on Building and Evaluating Resources for Biomedical Text Mining.

[8] Samanta P., Chaudhuri B.B. (2013). A simple real-word error detection and correction using local word bigram and trigram. Proceedings of the 25th Conference on Computational Linguistics and Speech Processing, ROCLING 2015.

[9] Grundkiewicz R., Junczys-Dowmunt M., Walker M., Ji H., Stent A. (2018). Near Human-Level Performance in Grammatical Error Correction with Hybrid Machine Translation. Proceedings of the 2018 Conference of the North American Chapter of the Association for Computational Linguistics: Human Language Technologies, Volume 2 (Short Papers).

[10] Zhao W., Wang L., Shen K., Jia R., Liu J., Burstein J., Doran C., Solorio T. (2019). Improving Grammatical Error Correction via Pre-Training a Copy-Augmented Architecture with Unlabeled Data. Proceedings of the 2019 Conference of the North American Chapter of the Association for Computational Linguistics: Human Language Technologies, Volume 1 (Long and Short Papers).

[11] Beloki Z., Saralegi X., Ceberio K., Corral A. (2020). Grammatical Error Correction for Basque through a seq2seq neural architecture and synthetic examples. Proces. Leng. Nat..

[12] Wilson B.J., Schakel A.M.J. (2015). Controlled Experiments for Word Embeddings. arXiv.

[13] Lai K.H., Topaz M., Goss F.R., Zhou L. (2015). Automated misspelling detection and correction in clinical free-text records. J. Biomed. Inform..

[14] Fivez P., Suster S., Daelemans W. (2017). Unsupervised Context-Sensitive Spelling Correction of Clinical Free-Text with Word and Character N-Gram Embeddings. Proceedings of the BioNLP 2017.

[15] Sutskever I., Vinyals O., Le Q.V., Ghahramani Z., Welling M., Cortes C., Lawrence N.D., Weinberger K.Q. (2014). Sequence to Sequence Learning with Neural Networks. Proceedings of the Advances in Neural Information Processing Systems 27: Annual Conference on Neural Information Processing Systems 2014.

[16] Vaswani A., Shazeer N., Parmar N., Uszkoreit J., Jones L., Gomez A.N., Kaiser L., Polosukhin I., Guyon I., von Luxburg U., Bengio S., Wallach H.M., Fergus R., Vishwanathan S.V.N., Garnett R. (2017). Attention is All you Need. Proceedings of the Advances in Neural Information Processing Systems 30: Annual Conference on Neural Information Processing Systems 2017.

[17] Peters M., Neumann M., Iyyer M., Gardner M., Clark C., Lee K., Zettlemoyer L., Walker M., Ji H., Stent A. (2018). Deep Contextualized Word Representations. Proceedings of the 2018 Conference of the North American Chapter of the Association for Computational Linguistics: Human Language Technologies, Volume 1 (Long Papers).

[18] Devlin J., Chang M.-W., Lee K., Toutanova K., Burstein J., Doran C., Solorio T. (2019). BERT: Pre-training of Deep Bidirectional Transformers for Language Understanding. Proceedings of the 2019 Conference of the North American Chapter of the Association for Computational Linguistics: Human Language Technologies, NAACL-HLT 2019.

[19] Liu Y., Ott M., Goyal N., Du J., Joshi M., Chen D., Levy O., Lewis M., Zettlemoyer L., Stoyanov V. (2019). RoBERTa: A Robustly Optimized BERT Pretraining Approach. arXiv.

[20] Bojanowski P., Grave E., Joulin A., Mikolov T. (2017). Enriching Word Vectors with Subword Information. Trans. Assoc. Comput. Linguist..

[21] Pennington J., Socher R., Manning C., Moschitti A., Pang B., Daelemans W. (2014). Glove: Global Vectors for Word Representation. Proceedings of the 2014 Conference on Empirical Methods in Natural Language Processing (EMNLP).

[22] Mikolov T., Chen K., Corrado G., Dean J., Bengio Y., LeCun Y. (2013). Efficient Estimation of Word Representations in Vector Space. Proceedings of the 1st International Conference on Learning Representations, ICLR 2013.

[23] Yin F., Long Q., Meng T., Chang K.-W., Jurafsky D., Chai J., Schluter N., Tetreault J.R. (2020). On the Robustness of Language Encoders against Grammatical Errors. Proceedings of the 58th Annual Meeting of the Association for Computational Linguistics.

[24] Alamri M., Teahan W. (2019). Automatic Correction of Arabic Dyslexic Text. Computers.

[25] Ferraro G., Nazar R., Alonso Ramos M., Wanner L. (2014). Towards advanced collocation error correction in Spanish learner corpora. Lang. Resour. Eval..

[26] Cho K., van Merrienboer B., Gulcehre C., Bahdanau D., Bougares F., Schwenk H., Bengio Y., Moschitti A., Pang B., Daelemans W. (2014). Learning Phrase Representations using RNN Encoder–Decoder for Statistical Machine Translation. Proceedings of the 2014 Conference on Empirical Methods in Natural Language Processing (EMNLP).

[27] Bahdanau D., Cho K., Bengio Y. (2015). Neural Machine Translation by Jointly Learning to Align and Translate. arXiv.

[28] Reese S., Boleda G., Cuadros M., Padró L., Rigau G., Calzolari N., Choukri K., Maegaard B., Mariani J., Odijk J., Piperidis S., Rosner M., Tapias D. (2010). Wikicorpus: A Word-Sense Disambiguated Multilingual Wikipedia Corpus. Proceedings of the International Conference on Language Resources and Evaluation, LREC 2010.

[29] Atserias J., Casas B., Comelles E., González M., Padró L., Padró M., Calzolari N., Choukri K., Gangemi A., Maegaard B., Mariani J., Odijk J., Tapias D. (2006). FreeLing 1.3: Syntactic and semantic services in an open-source NLP library. Proceedings of the Fifth International Conference on Language Resources and Evaluation, LREC 2006.

[30] Agirre E., Soroa A., Lascarides A., Gardent C., Nivre J. (2009). Personalizing PageRank for Word Sense Disambiguation. Proceedings of the EACL 2009 12th Conference of the European Chapter of the Association for Computational Linguistics.

[31] Miranda-Escalada A., Gonzalez-Agirre A., Armengol-Estapé J., Krallinger M., Cappellato L., Eickhoff C., Ferro N., Névéol A. (2020). Overview of Automatic Clinical Coding: Annotations, Guidelines, and Solutions for non-English Clinical Cases at CodiEsp Track of CLEF eHealth 2020. Proceedings of the Working Notes of CLEF 2020—Conference and Labs of the Evaluation Forum.

[32] Intxaurrondo A., Marimon M., Gonzalez-Agirre A., López-Martin J.A., Rodriguez H., Santamaria J., Villegas M., Krallinger M., Rosso P., Gonzalo J., Martinez R., Montalvo S., de Albornoz J.C. (2018). Finding Mentions of Abbreviations and Their Definitions in Spanish Clinical Cases: The BARR2 Shared Task Evaluation Results. Proceedings of the Third Workshop on Evaluation of Human Language Technologies for Iberian Languages (IberEval 2018) Co-Located with 34th Conference of the Spanish Society for Natural Language Processing (SEPLN 2018).

[33] Řehůřek R., Sojka P. (2010). Software Framework for Topic Modelling with Large Corpora. Proceedings of the LREC 2010 Workshop New Challenges for NLP Frameworks.

[34] Bird S., Calzolari N., Cardie C., Isabelle P. (2006). NLTK: The Natural Language Toolkit. Proceedings of the ACL 2006 21st International Conference on Computational Linguistics and 44th Annual Meeting of the Association for Computational Linguistics.

[35] Klein G., Kim Y., Deng Y., Senellart J., Rush A., Bansal M., Ji H. (2017). OpenNMT: Open-Source Toolkit for Neural Machine Translation. Proceedings of the 55th Annual Meeting of the Association for Computational Linguistics, ACL 2017.

[36] Yuan Z., Felice M., Ng H.T., Tetreault J.R., Wu S.M., Wu Y., Hadiwinoto C. (2013). Constrained Grammatical Error Correction using Statistical Machine Translation. Proceedings of the Seventeenth Conference on Computational Natural Language Learning: Shared Task, CoNLL 2013.

[37] Ooms J. The Hunspell Package: High-Performance Stemmer, Tokenizer, and Spell Checker for R. https://cran.r-project.org/web/packages/hunspell/vignettes/intro.html.

[38] Wang C., Cho K., Gu J. Neural Machine Translation with Byte-Level Subwords. Proceedings of the Thirty-Fourth AAAI Conference on Artificial Intelligence, AAAI 2020, the Thirty-Second Innovative Applications of Artificial Intelligence Conference, IAAI 2020, the Tenth AAAI Symposium on Educational Advances in Artificial Intelligence, EAAI 2020.

[39] Cardellino C. Spanish Billion Words Corpus and Embeddings. https://crscardellino.github.io/SBWCE/.

[40] Cañete J., Chaperon G., Fuentes R., Ho J.-H., Kang H., Pérez J. Spanish Pre-Trained BERT Model and Evaluation Data. Proceedings of the PML4DC at ICLR 2020.

[41] García-Díaz J.A., Cánovas-García M., Valencia-García R. (2020). Ontology-driven aspect-based sentiment analysis classification: An infodemiological case study regarding infectious diseases in Latin America. Future Gener. Comput. Syst..

[42] García-Díaz J.A., Cánovas-García M., Colomo-Palacios R., Valencia-García R. (2021). Detecting misogyny in Spanish tweets. An approach based on linguistics features and word embeddings. Future Gener. Comput. Syst..

[43] Apolinario-Arzube Ó., García-Díaz J.A., Medina-Moreira J., Luna-Aveiga H., Valencia-García R. (2020). Comparing Deep-Learning Architectures and Traditional Machine-Learning Approaches for Satire Identification in Spanish Tweets. Mathematics.

[44] Lee J., Yoon W., Kim S., Kim D., Kim S., So C.H., Kang J. (2020). BioBERT: A pre-trained biomedical language representation model for biomedical text mining. Bioinformatics.

[45] García-Sánchez F., Colomo-Palacios R., Valencia-García R. (2020). A social-semantic recommender system for advertisements. Inf. Process. Manag..

